# Transcriptional elongation requires DNA break-induced signalling

**DOI:** 10.1038/ncomms10191

**Published:** 2015-12-16

**Authors:** Heeyoun Bunch, Brian P. Lawney, Yu-Fen Lin, Aroumougame Asaithamby, Ayesha Murshid, Yaoyu E. Wang, Benjamin P. C. Chen, Stuart K. Calderwood

**Affiliations:** 1 Department of Radiation Oncology, Beth Israel Deaconess Medical Center, Harvard Medical School, Boston, Massachusetts 02115, USA; 2 Center for Cancer Computational Biology, Dana Farber Cancer Institute, Boston, Massachusetts 02130, USA; 3 Department of Radiation Oncology, University of Texas Southwestern Medical Center, Dallas, Texas 75390, USA

## Abstract

We have previously shown that RNA polymerase II (Pol II) pause release and transcriptional elongation involve phosphorylation of the factor TRIM28 by the DNA damage response (DDR) kinases ATM and DNA-PK. Here we report a significant role for DNA breaks and DDR signalling in the mechanisms of transcriptional elongation in stimulus-inducible genes in humans. Our data show the enrichment of TRIM28 and γH2AX on serum-induced genes and the important function of DNA-PK for Pol II pause release and transcriptional activation-coupled DDR signalling on these genes. γH2AX accumulation decreases when P-TEFb is inhibited, confirming that DDR signalling results from transcriptional elongation. In addition, transcriptional elongation-coupled DDR signalling involves topoisomerase II because inhibiting this enzyme interferes with Pol II pause release and γH2AX accumulation. Our findings propose that DDR signalling is required for effective Pol II pause release and transcriptional elongation through a novel mechanism involving TRIM28, DNA-PK and topoisomerase II.

## Abstract (web summary)

RNA polymerase II (Pol II) pause release and transcriptional elongation involve phosphorylation of TRIM28 by the DNA damage response (DDR) kinases. Here, Bunch *et al*. show that DDR signalling is coupled with and required for transcriptional elongation in stimulus-inducible genes and involves topoisomerase II.

## Graphical abstract

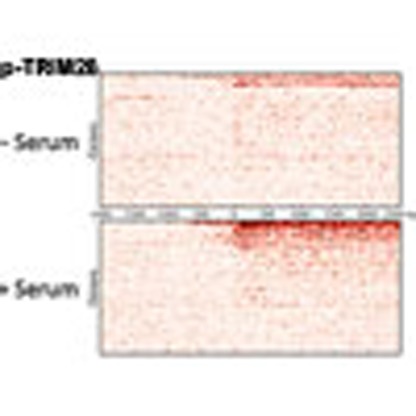

Regulation of transcription is a crucial mechanism for the development and survival of cellular organisms through appropriate control of genetic readout. Loss of such control thwarts proper organismal development and homeostasis. To achieve fine-tuning in gene expression, each of transcriptional stages, including initiation, elongation and termination, is tightly controlled by various protein and nucleic acid factors. In addition to these regulatory events, recent genome-wide analyses have indicated another important regulatory stage, known as RNA polymerase II (Pol II) promoter proximal pausing as a widespread mechanism to regulate gene expression^1^,^2^,^3^,^4^,^5^,^6^,^7^. Engaging Pol II at the promoter-proximal site before processive elongation appears to be a preparative step, whereby genes can be primed for rapid induction, assuring prompt and decisive cell regulation^8^,^9^.

Although the mechanisms of Pol II pausing and pause release are incompletely understood, several transcription factors have been shown to regulate these processes. DSIF and NELF induce and stabilize pausing^10^, while TFIIS^2^, Myc and positive transcription elongation factor b (P-TEFb) help release Pol II from the pausing site^1^. P-TEFb phosphorylates DSIF, NELF and the C-terminal domain of Pol II (Pol II CTD), permitting pause release^11^. Our previous studies indicated TRIM28 to be another regulator of promoter proximal pausing in mammalian cells^12^. We showed that the factor TRIM28 is associated with the Pol II pause site at a model paused gene, *HSPA1B* (human *HSP70-2* gene), and stabilizes Pol II pausing, thus suppressing elongation. TRIM28 knockdown increased Pol II occupancy in the gene body at a number of genes, suggesting that TRIM28 regulates Pol II elongation genome wide^12^. In addition, pause release and processive elongation at *HSPA1B* involved the phosphorylation of TRIM28 at S824 by ataxia telangiectasia mutated (ATM) and DNA-dependent protein kinase (DNA-PK)^12^,^13^.

Interestingly, some of the features TRIM28-mediated regulation of pausing are reminiscent of DNA damage repair signalling processes: it has been shown that TRIM28 is recruited rapidly to DNA lesions and becomes phosphorylated at S824 by ATM and DNA-PK, thus facilitating DNA repair^14^,^15^. We therefore hypothesized that the TRIM28 phosphorylation at *HSPA1B* may indicate the involvement of DNA damage response (DDR) signalling during Pol II pause release and transcriptional activation. Quite a few previous studies indeed supported this hypothesis. Recent *in vitro* and *in vivo* studies suggested that DNA torsion generated by elongating RNA polymerases may be involved in Pol II stalling^16^,^17^. Negative supercoiling in the upstream of an elongating Pol II, which could lead to R-loop formation, is known to be resolved by topoisomerase I^18^, indicating a requirement for reduction of DNA torsion during transcriptional elongation. In addition, it was shown that inhibition of topoisomerases decreases expression of longer transcripts in yeast^19^,^20^. Also in yeast, a transcriptionally more active strain produces more spontaneous mutations than less active variants^21^, implying DNA break/repair events that may account for the high mutation rate during active transcription. In agreement with these findings, DNA strand break loci have been mapped more frequently within or near transcriptionally active regions of genes than non-transcribed regions, suggesting a positive relationship between transcriptional activity and DNA strand breaks^18^,^22^.

In this study, our findings indicate the coupling and requirement of DNA double-strand breaks (DSBs)/DDR signalling with transcriptional activation and elongation in stimulus-inducible protein-coding genes in humans. We show that DDR proteins such as phosphorylated TRIM28 (S824), activated DNA-PK complex and γH2AX are accumulated during Pol II pause release in the transcription start sites (TSSs) of these genes. DDR signalling occurs throughout transcriptional elongation during gene induction, as evidenced by phosphorylated TRIM28 (S824) and γH2AX on the actively transcribing units and by co-localization of Pol II phosphorylated at the CTD serine 2 (S2 Pol II, a bona fide indicator of processive elongation) with activated DNA-PK. Strikingly, our data reveal significant roles of DNA-PK in transcriptional elongation because inhibition of this factor interferes with Pol II pause release and markedly reduces S2 Pol II in activated paused genes. We also show that DDR signalling results from active transcriptional elongation because inhibition of P-TEFb, a kinase that phosphorylates S2 of Pol II CTD, reduces the level of γH2AX in these genes. Like canonical DDR signalling induced by random or targeted DNA breaks, H2AX is phosphorylated by DNA-PK (probably also by ATM) during transcription-coupled DDR signalling, as indicated by the reduced level of γH2AX on actively transcribing units in the presence of DNA-PK inhibitor. In addition, our data propose the critical roles of topoisomerase II to mediate DSBs for Pol II pause release and transcriptional elongation. Our assays show that topoisomerase II is recruited on the activated paused genes, and inhibiting this factor using a small molecule leads to retention of Pol II in TSSs and reduces the levels of Pol II, S2 Pol II and γH2AX in the gene bodies of these genes, interfering with both Pol II pause release and processive elongation.

## Results

### γH2AX accumulation upon transcriptional activation

It has been thought that DNA strand break occurrence during transcription could be caused by some of the consequences of transcriptional stresses such as transcription–replication collision^23^ or R-loop formation^18^. However, scheduled DNA breaks, for example, oestrogen or androgen receptor-inducible DSB mediated by topoisomerase IIβ (TOPIIB), have been also reported in the promoters of transcribing genes in human cells^24^,^25^,^26^,^27^. These findings could imply the advantages or functional necessities of DNA strand break to increase transcriptional potential in activated genes.

We therefore aimed to determine whether DDR signalling might be induced upon transcriptional activation at inducible genes containing paused Pol II in human cells. First, we questioned whether TRIM28 phosphorylation by DNA-PK and ATM at activated *HSPA1B*^12^ might be an indicator of DDR signalling triggered by transcription. H2AX is a variant form of histone H2A, and ATM and DNA-PK phosphorylate Ser139 on H2AX during DNA damage, generating γH2AX, a bona fide indicator of DDR signalling^28^,^29^,^30^.

**Figure 1:**
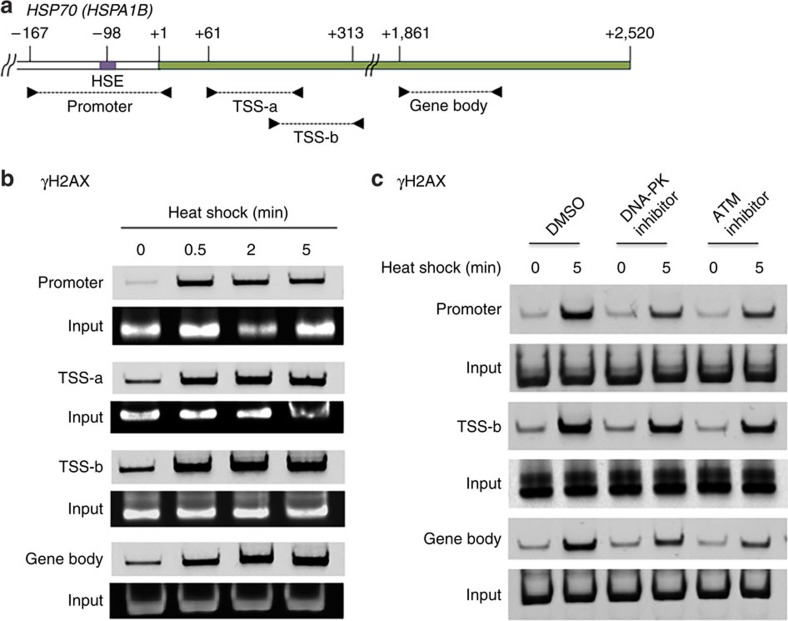
γH2AX accumulation at *HSPA1B* upon transcriptional activation. (**a**) *HSPA1B* promoter, TSS and gene body with marked primers (arrowheads) used in ChIP–PCR. HSE, heat-shock element (HSF1-binding site). (**b**) ChIP–PCR showing association of γH2AX with the *HSPA1B* promoter, TSS and gene body upon heat shock to activate transcription. (**c**) ChIP–PCR displaying the effect of DNA-PK and ATM inhibitors on γH2AX accumulation at *HSPA1B*.

Phosphorylation of H2AX was examined at *HSPA1B* using chromatin immunoprecipitation (ChIP)–PCR analysis in human embryonic kidney 293 (HEK293) cells (Fig. 1). Transcriptional activation by heat shock led to the accumulation of γH2AX at *HSPA1B*, including sequences adjacent to the TSS (–167 to +10, promoter; +61 to +313, TSS-a and TSS-b) and through the gene body (gene body, +1,861 to +2,010) near the 3′-end of the gene (Fig. 1a,b; Supplementary Fig. 1). This effect was rapid, established within 30 s of heat shock (Fig. 1b; Supplementary Fig. 1), a similar time course to HSF1 binding and *HSP* transcriptional activation^12^,^31^. Since DNA-PK and ATM phosphorylate γH2AX during DDR, we asked whether these same kinases were responsible for H2AX phosphorylation during *HSPA1B* activation. γH2AX accumulation was effectively inhibited by small molecules, NU7441 or KU55933, targeting DNA-PK or ATM (Fig. 1c). These results suggested the potential occurrence of DNA break and triggering of DDR signalling, involving pTRIM28, DNA-PK, ATM and γH2AX, at the TSS and gene body of *HSPA1B* during transcriptional activation.

### Coupling of DDR signalling with transcriptional activation

**Figure 2:**
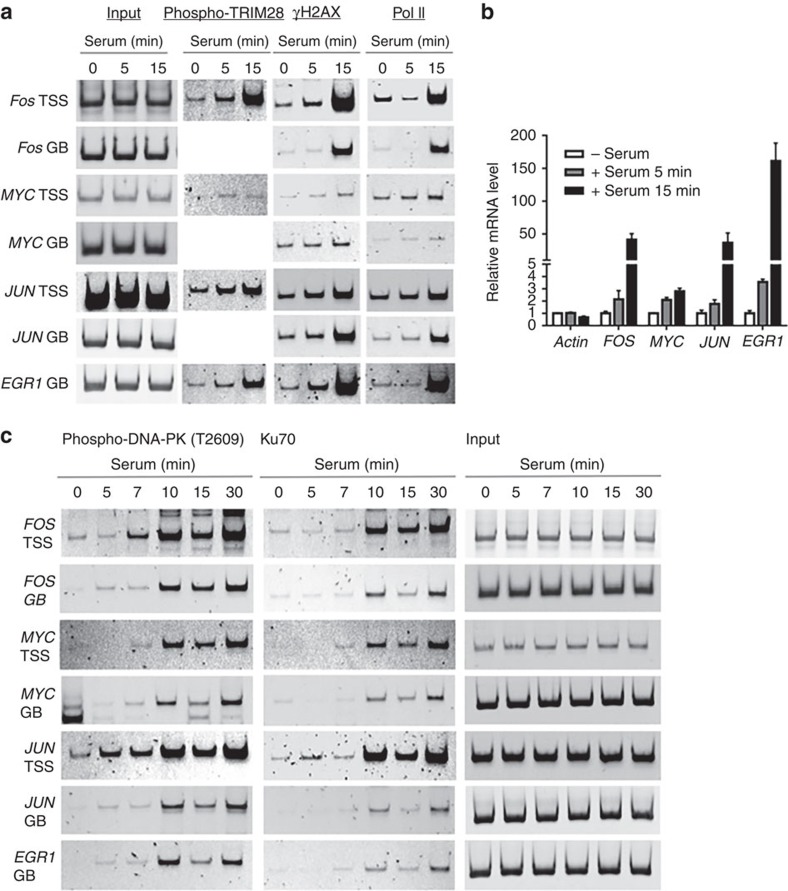
DNA repair proteins are accumulated upon transcriptional activation. (**a**) ChIP–PCR showing phospho-TRIM28 (S824) and γH2AX accumulation in the TSSs and gene bodies (GBs) of immediate-early genes such as *JUN*, *MYC*, *EGR1* and *FOS* upon serum-induced transcriptional activation. Simultaneous ChIP–PCR analysis of Pol II shows the increase Pol II occupancy, thus verifying transcriptional activation at *JUN*, *MYC*, *EGR1* and *FOS* upon serum induction at the given time points. (**b**) qRT–PCR analysis showing mRNA expression of *JUN*, *EGR1*, *FOS* and *MYC* in control (serum−) and serum-induced samples (serum 5 min and serum 15 min). *β-Actin* was included as a negative control for serum induction and as an internal control for qRT–PCR. Error bars represent s.d. after normalization with −serum (for each bar, *P* value<0.05; *n*⩾3). (**c**) ChIP–PCR data showing phospho-DNA-PK (T2609; an activating phosphorylation in DDR signalling) and Ku70 accumulation at the immediate-early genes upon transcriptional activation.

To rule out the possibility that activation of DDR signalling in the heat-shock system might result directly from thermal stress, we utilized another mild, non-genotoxic transcriptional induction system—serum-inducible gene expression. HEK293 cells were synchronized in G_0_ using 0.1% serum for 17.5 h and then replenished with 18% serum for 5 or 15 min to activate entry into the cell cycle and the transcription of immediate-early genes such as *JUN*, *FOS*, *EGR1* and *MYC*. While serum addition did not change total TRIM28 level, this stimulus led to significant accumulation of pTRIM28 at *JUN*, *FOS*, *EGR1* and *MYC* genes (Fig. 2a; Supplementary Fig. 2). Consistently, γH2AX was accumulated both at the TSSs and within gene bodies of these genes, similarly to its enrichment upon *HSPA1B* activation (Figs 1b and 2a; Supplementary Fig. 1). In addition, the quantitative PCR with reverse transcription (RT–qPCR) analysis showed increases in gene expression of these immediate-early genes, in a similar pattern to Pol II occupancy monitored by ChIP–PCR (Fig. 2a,b).

Our previous studies indicated that TRIM28 stabilizes Pol II pausing, an effect reversed through transcription-activated phosphorylation downstream of DNA-PK. We also showed in proteomic studies that TRIM28 interacts with the DNA-PK catalytic subunit (DNA-PKcs) and Ku70 (XRCC6), a component of the DNA-PK complex that associates with the broken ends of duplex DNA along with its partner Ku80 (ref. 12). Therefore, we next examined whether these representative DNA repair proteins would be co-recruited along with pTRIM28 and γH2AX upon transcriptional activation. Specifically, we examined recruitment of an activated form of DNA-PKcs, phosphorylated at pT2609, a residue modified exclusively by ATM during DNA damage^32^. Levels of pDNA-PKcs (T2609) and Ku70 on the *JUN*, *FOS*, *EGR1* and *MYC* genes were minimal in uninduced cells but accumulated in a similar time course to Pol II increase in serum-induced cells (Fig. 2a,c). It is important to note that the examined DDR factors were recruited to the regions between +121 and +315 and between +2,471 and 2,940 (for *Myc*, +4,497 and 4,663) of these representative paused genes, indicating an apparent coupling between DDR signalling and transcriptional elongation. These results suggested that, as shown for *HSPA1B* activation, DDR signalling was triggered during transcriptional activation and associated with transcriptional elongation at these stimulus-inducible genes.

**Figure 3:**
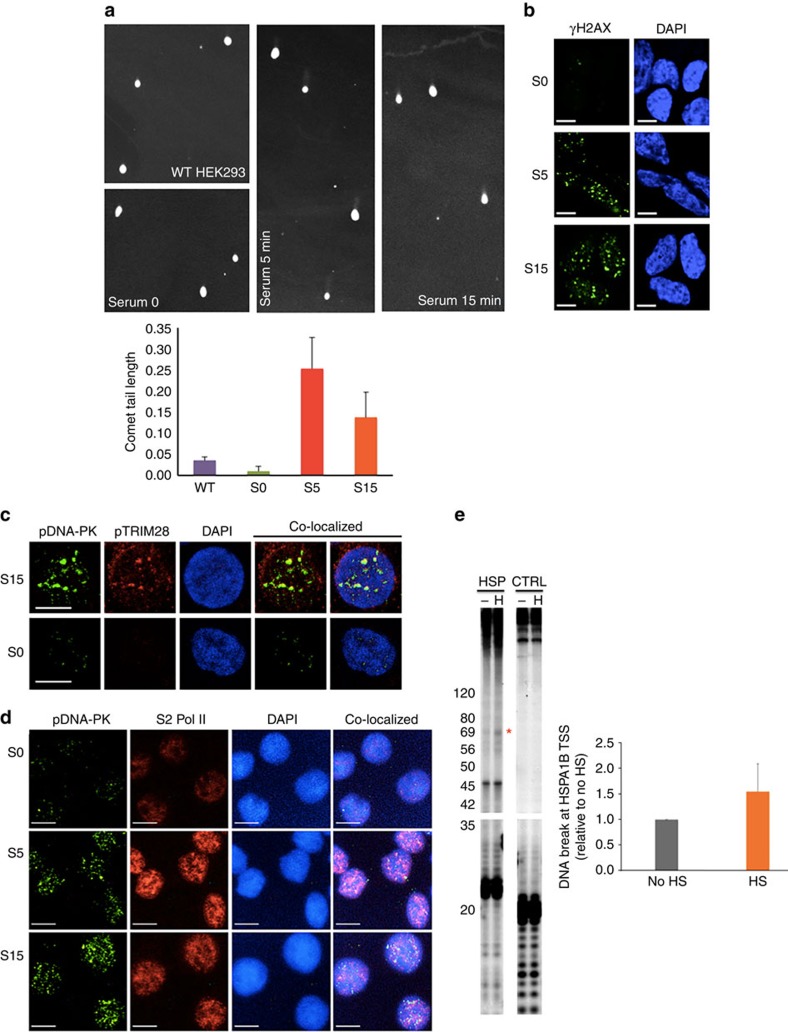
DNA break occurrence upon transcriptional activation in cellular and molecular levels. (**a**) Comet assay results visualizing cellular tail smears which indicate single- or double-stranded DNA break events upon serum induction (upper panel). Bar graphs showing comet tail quantification using ImageJ (bottom panel, average tail length with s.d., *n*⩾6). WT, non-serum-deprived control; S0, serum-deprived control; S5, serum induced for 5 min after serum deprivation; S15, serum induced for 15 min after serum deprivation. (**b**) Immunofluorescence displaying the increased levels of cellular γH2AX foci upon serum induction. A scale bar on the bottom, left of each image indicates 5 μm. (**c**) Immunofluorescence showing pTRIM28 increase and co-localization with pDNA-PK (T2609) upon serum-induced transcriptional activation (scale bar, 10 μm). (**d**) Immunofluorescence showing co-localization of S2 Pol II and pDNA-PK (T2609), visualizing a correlation between transcriptional elongation and DDR signalling upon serum induction (scale bar, 10 μm). (**e**) A DNA break locus at around +69 of the *HSPA1B* template DNA upon transcriptional activation. HSP, *HSPA1B*; CTRL and *GAPDH* as a control; –, non-heat-shocked; H, heat-shocked for 5 min. Size markers are shown on the left of the gel in base-pairs. A band indicating a DNA break event that became prominent upon heat-shock-induced *HSPA1B* was marked with a red star. Bar graphs quantifying the intensity of the band (average with standard deviation; *n*=3 cell cultures). HS, heat-shocked HEK293.

Since the presence of γH2AX, pDNAPK and Ku70 suggested triggering of DDR signalling during transcription, we next investigated whether DNA strand breaks could be visualized using the Comet assay. While small Comet tails could be observed in control HEK293 cells, the appearance of detectable Comet tails, implying single- or double-stranded DNA breaks, increased in serum-induced cells (Fig. 3a; Supplementary Fig. 3a). We therefore next examined another index of DNA damage, carrying out immunofluorescence analysis of γH2AX foci, characteristic sequela of DNA DSB and repair. Rapid formation of γH2AX foci was detected in serum-induced and also heat-shocked HEK293 cells (Fig. 3b; Supplementary Figs 3b–c).

In addition, increased levels of pDNA-PKcs (T2609) and pTRIM28 and the co-localization of these proteins were shown upon transcriptional activation (Fig. 3c). Importantly, upon serum induction, pDNA-PKcs (T2609) appeared to be co-localized with S2 Pol II, a form of Pol II essential for processive elongation (Fig. 3d; Supplementary Figs 4a–b). Another DDR-activated phosphorylation site of DNA-PKcs, T2647, a residue whose phosphorylation is dependent on ATM during DNA damage^32^, also became rapidly phosphorylated and co-localized with S2 Pol II upon serum induction (Supplementary Fig. 4c). These data suggested that DNA breaks occurred during transcriptional activation and that H2AX, TRIM28 and DNA-PKcs were coordinately phosphorylated downstream of ATM on actively transcribing genes.

Since DDR signalling was visualized at TSSs of established paused genes during Pol II pause release (Figs 1 and 2), we attempted to locate a DNA break site(s) at the *HSPA1B* TSS using primer extension assay^25^. We chose *HSPA1B* since the pausing site of this gene was shown to be near +70 from TSS in a previous study^12^, and DDR proteins were recruited near the pausing site upon Pol II pause release (Fig. 1)^12^. An increased band appeared near +69 from the TSS on the *HSPA1B* template DNA in heat-shocked HEK293 cells, suggesting a DNA break at this locus upon transcriptional activation (Fig. 3e). We observed pre-existing bands at the promoter-proximal region of the *HSPA1B* non-template DNA in non-heat-shocked, control samples, which obscured detection of an increased band upon heat shock (Supplementary Fig. 5). These results suggested a potential DNA break on the template DNA upon pause release and the susceptibility for DNA break of non-template DNA, as it has been previously reported^18^,^33^, at the Pol II pausing site of *HSPA1B*.

### Coupling of DDR signalling with elongation genome wide

Next, we asked whether the DDR signalling proteins observed at selected genes could be detected at diverse, transcriptionally activated genes in genome-wide analysis. pDNA-PK (T2609), pTRIM28, γH2AX and Pol II were monitored by ChIP sequencing (ChIP-seq) analysis. Transcriptional activation at multiple genes was induced by serum in HEK293 cells, indicated by increased Pol II occupancy.

**Figure 4:**
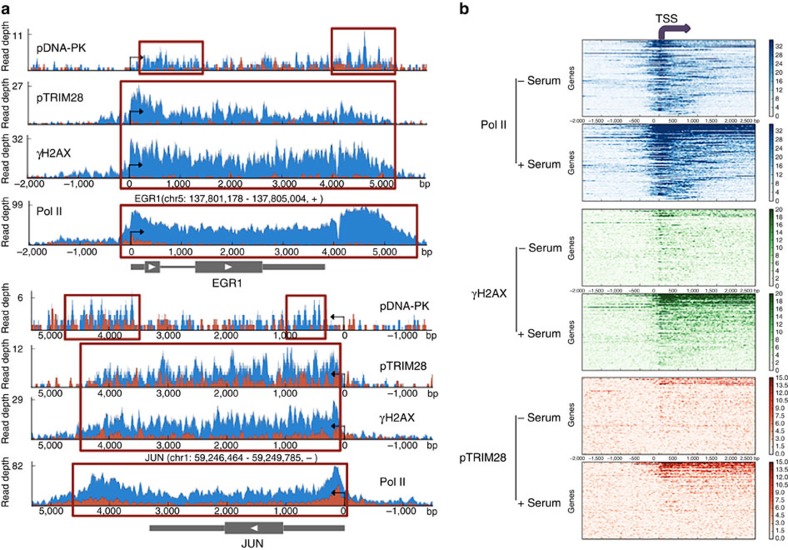
ChIP-seq analysis of Pol II, γH2AX, pTRIM28 (S824) and pDNA-PK (T2609) in representative and a number of early response genes. (**a**) Chromosome views of Pol II, γH2AX, pTRIM28 (S824) and pDNA-PK (T2609) at representative serum inducible genes such as *EGR1* (top) and *JUN* (bottom). Blue peaks for serum-induced cells and red peaks for control, non-serum-induced cells. Peaks appearing upon serum induction were marked with red boxes. (**b**) Heat maps of Pol II (blue), γH2AX (green) and pTRIM28 (S824; red) at serum-induced genes sorted by Pol II gene body occupancy upon serum induction (*n*=100). TSS is at 0 and also marked with a purple arrow. Control cells, −serum; 15 min serum-induced cells, +serum.

Known immediate-early genes such as *JUN*, *FOS* and *EGR1* and the early serum response gene, *ATF3* (ref. 34) showed representative Pol II pausing profiles at TSSs in the uninduced state and then underwent marked increases in Pol II gene body occupancy upon serum induction (Fig. 4a; Supplementary Figs 6a–b). Significantly, pTRIM28 and γH2AX increased throughout the transcribing region of these genes upon serum induction, while the levels of the phosphoproteins were much lower in the control, uninduced genes, (Fig. 4a; Supplementary Fig. 6a). Distinct from the observations for γH2AX and pTRIM28, pDNA-PK (T2609) peaks were detected in the proximity of the TSS and at the gene termini of *EGR1*, *JUN* and *FOS* (Fig. 4a; Supplementary 6a). In addition, transcriptional activation-induced γH2AX accumulation appeared to be concentrated only within the transcribed units, without spreading outside these gene boundaries (Fig. 4a). We note that this phenomenon is different from the one reported for γH2AX accumulation by random or targeted DSB induced with DNA-damaging agents or a sequence-specific enzyme. In these cases, γH2AX spreads distances of kilo (yeast) to mega bases (mammals) in both directions from the DSB locus^35^,^36^,^37^. These results showed that DDR proteins are activated and DDR signalling occurs in the gene body of established paused genes during transcriptional elongation. Consistent with our findings, it was recently reported that γH2AX is accumulated on activated early-response genes, proportional to the sizes of genes in the neuronal cells^38^. In particular, the finding that pTRIM28 and γH2AX accumulation follows a similar trend to Pol II in the transcribing units strongly suggested the significant role of these factors in transcriptional elongation.

To examine Pol II pausing changes upon serum induction, we defined TSS and TSS-proximal windows as start or end ±150, respectively, and defined gene body window as +250 to the gene end. Pausing indices of coding genes with a ChIP peak and sufficient gene body reads (read density>0.05, *n*=1,588) were compared between control and serum-induced cells. A set of genes including known serum response genes such as *JUN*, *FOS* and *EGR1* displayed pausing indices with at least 50% reduction for increased Pol II gene body occupancy upon serum induction (*n*=285). Strikingly, pTRIM28 and γH2AX occupancy became enriched throughout TSSs and gene bodies at a number of genes with increased Pol II occupancy (−2,000, +2,500) upon serum induction (*n*=100, Fig. 4b). We noted that γH2AX occupancy was increased within the boundaries of transcription units, in a manner similar to the Pol II occupancy increase observed on transcribed regions in serum-induced cells (Fig. 4b). As mentioned above, it is notable that this pattern of γH2AX increase is distinct from one caused by DSB resulting from targeted digestions or genotoxic stresses^35^,^36^,^37^. It is interesting to note that a recent study indicated an overlap between topoisomerase II and CCCTC-binding factor (CTCF)-binding sites, suggesting a potential function of CTCF to draw the boundaries of DDR in the gene activation involving topoisomerase II-mediated DSBs^38^. Overall, these results confirmed DDR signalling to be coupled with transcriptional elongation and the proteins involved in such signalling to be activated and engaged with many inducible genes upon transcriptional activation in human cells.

### DNA-PK is required for Pol II pause release and elongation

**Figure 5:**
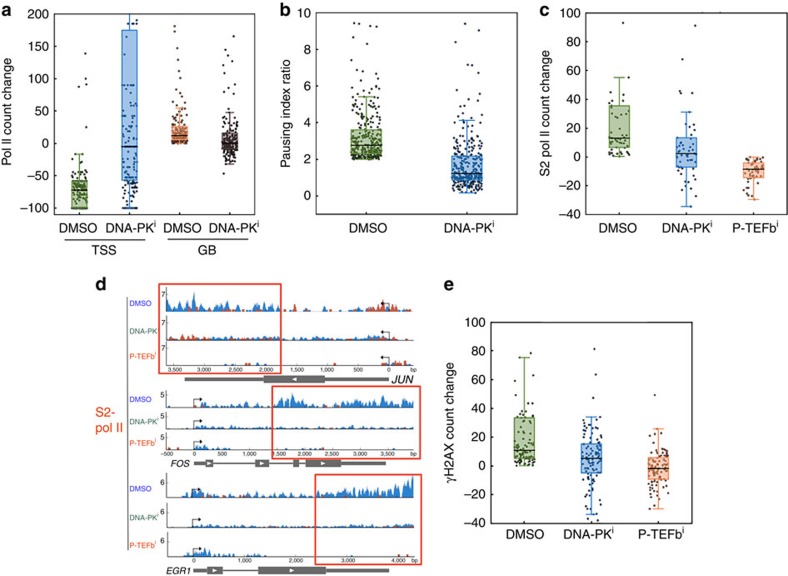
DDR signalling is coupled with and required for effective Pol II pause release and elongation. (**a**) Comparison of Pol II occupancy changes between non-serum-induced and serum-induced cells in DMSO (control), DNA-PK^i^ (DNA-PKcs inhibited) and P-TEFb^i^ (P-TEFb inhibited) conditions. Transcriptionally activated genes, with pausing index decrease over twofold upon serum induction, were included to measure Pol II occupancy change in the TSSs and gene bodies (GBs; *n*=211). Normalized Pol II count change was shown in percentage (TSS, *P*<0.0001, confidence interval (–63.8, –86.0); GB, *P*<0.0001, confidence interval (–15.6, –8.5)). (**b**) Box plots showing pausing index ratio of transcriptionally activated genes between non-serum-induced, control and serum-induced cells with or without DNA-PKcs inhibition (DNA-PK^i^; *n*=301, *P*<0.0001, confidence interval (1.32, 1.73)). DNA-PK function is important for Pol II pause release. (**c**) Box plots of S2 Pol II occupancy percentage changes between non-serum-induced and serum-induced cells in the presence of DMSO (control), DNA-PK^i^ and P-TEFb^i^ (*n*=57, *P* value<0.0001, confidence interval (5.4, 24.9) for DMSO and DNA-PK^i^, (18.5, 36.0) for DMSO and CDK9^i^, (7.4, 16.5) for DNA-PK^i^ and CDK9^i^). (**d**) Chromosome views of *JUN*, *FOS* and *EGR1* showing S2 Pol II occupancy changes in the presence of DNA-PKcs and P-TEFb inhibition. The colour code is identical to the one used for Fig. 4a. DNA-PKcs inhibition markedly reduced S2 Pol II occupancy increase in the gene body (marked with red boxes), during transcriptional activation. (**e**) Box plots of γH2AX occupancy changes in percentage between non-serum-induced and serum-induced cells in the presence of DMSO (control), DNA-PK^i^ and P-TEFb^i^ (*n*=108). The data showed that γH2AX increase upon serum induction is positively regulated by DNA-PK (*P*=0.0036, confidence interval (1.4, 10.3)) and P-TEFb (*P*<0.0001, confidence interval (9.1, 17.1)). For DNA-PK^i^ and CDK9^i^, *P* value<0.0001, confidence interval (3.7, 10.6).

Next, we wanted to verify whether DDR signalling accompanied by transcriptional activation is necessary for Pol II pause release and processive elongation. To answer this question, DNA-PKcs was inhibited using NU7441 for an hour in HEK293 cells, and cells were induced by serum exposure for 15 min (S15). Pol II occupancy and pausing indices were then compared between wild type (WT) and DNA-PKcs-inhibited HEK293 cells through the genomic analysis. DNA-PKcs inhibition interfered with the increase of Pol II occupancy in the gene body at transcriptionally activated genes (categorized through gene body Pol II increase and pausing index decrease over twofold upon serum induction in the dimethylsulphoxide (DMSO) condition; *n*=211 genes; Fig. 4c). On the other hand, Pol II occupancy was noticeably increased in the TSSs in the presence of DNA-PKcs inhibitor at these genes (Fig. 5a). We also note that DNA-PKcs inhibition appeared to increase Pol II occupancy in the TSSs of serum-uninduced cells, compared with DMSO control. These observations could imply that DNA-PK might suppress transcriptional initiation as proposed for ribosomal RNA transcription^39^ in a previous study (Supplementary Fig. 7). However, in spite of increase of TSS Pol II accumulation, Pol II became ineffectively progressed into the gene body in the presence of DNA-PKcs inhibitor, indicating that the function of DNA-PK is important for Pol II elongation upon transcriptional activation.

Pausing index comparison between control and S15 cells further confirmed the function of DNA-PK in Pol II pause release and elongation. Pausing index, a ratio of Pol II occupancy between the TSS and gene body (TSS Pol II/gene body Pol II), was calculated to measure the degree of Pol II pausing^12^,^40^. As described above, TSS-proximal windows were defined as ±150 nt, and gene body windows were defined as +250 to the gene end. DNA-PKcs inhibition clearly reduced the pausing index ratio between serum-uninduced (S0) and -induced states (S15; pausing index in S0/pausing index in S15) at a number of transcriptionally activated genes (*n*=301, Fig. 5b). Together with the data shown in Fig. 5a, these results suggest that Pol II release from the TSSs upon transcriptional activation is deregulated in the presence of DNA-PKcs inhibitor, and thus that DNA-PK functions in Pol II pause release and transcriptional elongation.

Since Pol II pause release and processive elongation were relatively decreased in DNA-PKcs inhibition, we hypothesized that S2 Pol II, an established indicator for processive elongation, might be decreased in the same condition. To test this hypothesis, DNA-PKcs was inhibited by NU7441 in HEK293 cells, and the control and DNA-PKcs-inhibited cells were induced by serum. As a positive control, CDK9, a kinase subunit of P-TEFb, was inhibited to block Pol II CTD phosphorylation at S2 using flavopiridol. As shown in Fig. 5c, S2 Pol II level became decreased in a subset of the activated genes (*n*=57) in DNA-PKcs-inhibited cells, compared with the DMSO control. S2 Pol II occupancy change as a function of DNA-PKcs was also clearly shown in representative immediate early, paused genes, *FOS*, *JUN* and *EGR1* (Fig. 5d). Consistent with the Pol II data in Fig. 5a,b, these results assured the important function of DNA-PK in Pol II elongation.

In addition, we probed γH2AX occupancy changes at the activated genes upon serum induction, comparing DNA-PKcs- or CDK9-inhibited cells with WT. From the observations that DNA-PK facilitates Pol II pause release and processive elongation (Fig. 5a–d) and that γH2AX is accumulated on transcriptionally activated genes (Fig. 4b), dependent on DNA-PK and ATM (Fig. 1c), it was expected that the γH2AX level change would be reduced without active DNA-PKcs. Indeed, it was shown that DNA-PKcs inhibition lowered γH2AX occupancy increase upon serum induction in a subset of activated genes in comparison with WT (*n*=108; Fig. 5e). The reduction was mild, likely due to a partial effect, considering the fact that ATM could still phosphorylate H2AX^29^ in the absence of DNA-PK function in the cell as seen in Fig. 1c. Significantly, CDK9 inhibition, which interferes with Pol II pause release and elongation, reduced γH2AX occupancy increase upon transcriptional activation at these genes (Fig. 5e). This result confirmed a coupling between transcriptional elongation and DDR signalling, and suggested γH2AX accumulation as a consequence of transcriptional pause release and elongation.

### Topoisomerase II induces DSBs required for elongation

TOPIIB, an enzyme that produces DSB during its effects on the modulation of DNA topology, has been reportedly required for oestrogen and androgen target gene transcription^24^,^25^,^26^,^41^. In our oligo pull-down and mass spectrometry analyses, topoisomerase IIα and IIβ (TOPII) were abundantly associated with *HSPA1B* double-stranded DNA including the promoter and TSS (–467 to +216). Since DNA breaks were induced upon transcriptional activation and elongation and the transcription-coupled DNA breaks appeared to be distinctive from random ones, we asked whether topoisomerase II could mediate DSBs on productively transcribing genes.

**Figure 6:**
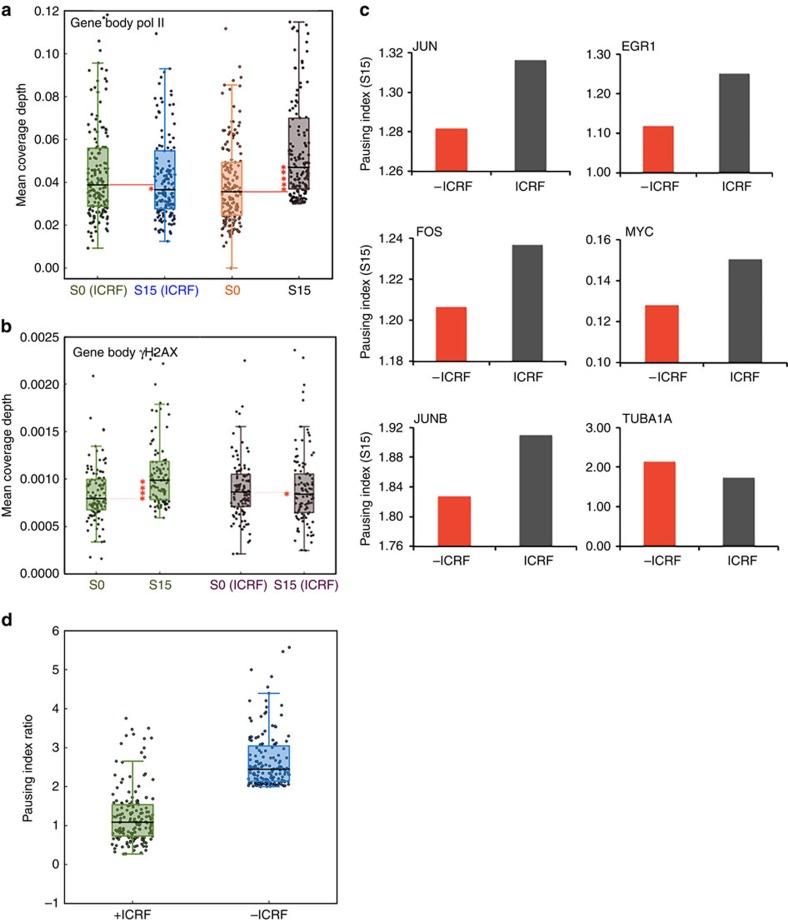
Topoisomerase II functions to induce DNA breaks and DDR signalling as a positive regulator of transcriptional elongation. (**a**) Comparison of mean coverage depth for gene body Pol II occupancy at a subset of transcriptionally activated genes from control cells, S0; control cells treated with ICRF193, S0 (ICRF); serum-induced cells, S15; and serum-induced cells treated with ICRF193, S15 (ICRF). Genes with pausing index decrease over two0fold upon serum induction with sufficient gene body-read density (>0.05) were collected and analysed (*n*=162). To assess the significance of greater mean coverage depth in the induced state, estimated one-sided *P* value were obtained via a bootstrap resampling procedure (*P* value=0.25 for S0 and S15 in the presence of ICRF193, confidence interval (–0.004, 0.007); *P* value<0.0001 for S0 and S15 in the absence of ICRF193, confidence interval (0.007, 0.18)). (**b**) Comparison of mean coverage depth for gene body γH2AX occupancy at a subset of activated genes (*n*=108) with or without topoisomerase II inhibitor (ICRF). *P* values are 0.0016 for S0 and S15 in DMSO control (confidence interval (6e−0.5, 0.0002)) and 0.6751 for S0 and S15 with ICRF193 (confidence interval (–0.0001, 7.3e−0.5)). (**c**) Pausing index change of known early immediate genes such as *JUN*, *EGR1*, *JUNB*, *IER5*, *FOS* and *MYC* in the presence and absence of ICRF193. Tubulin (*TUBA1A*) was shown as a non-paused, non-induced control gene. Pausing indices of these genes became increased in the presence of ICRF193 for reduced transcriptional activation, compared with the control without ICRF193. (**d**) Pausing index ratio of a subset of serum activated genes (*n*=162, *P* value<0.0001, confidence interval (1.2, 1.5)) between control and serum-induced cells in the presence (+ICRF) and absence (−ICRF) of ICRF193. Genes with low Pol II counts were excluded to prevent from misled high pausing indices. Genes with pausing index ratio change over 2 in control (−ICRF) were included in the comparison. Inhibition of TOPII-mediated DSB abolished pausing index decrease upon serum-induced transcriptional activation, suggesting an important function of TOPII for effective Pol II pause release.

Initially, we observed that TOPIIB occupancy became increased at established immediate-early genes upon serum induction in ChIP analysis (Supplementary Fig. 8). Therefore, the potential function of TOPII in transcriptional elongation was tested in serum-inducible genes, utilizing ICRF193, a specific inhibitor of the catalytic activity of TOPII^42^. While TOPII inhibition caused a mild increase in Pol II occupancy at TSSs, it abolished serum-induced Pol II occupancy increase in the gene bodies of a subset of serum-induced genes (genes with a ChIP-seq peak, sufficient gene body-read density (>0.05), and at least twofold pausing index decrease upon serum induction, *n*=162; Fig. 6a; Supplementary Fig. 9). In addition, TOPII inhibition reduced γH2AX occupancy increases in the gene bodies of activated genes (*n*=108, Fig. 6b; Supplementary Fig. 10), indicating that TOPII could regulate Pol II elongation in the serum-inducible genes. Significantly, the pausing indices of these genes, a cohort that included the established immediate-early-response genes, changed to a markedly lesser extent or even increased upon serum induction in the presence of ICRF193 compared with control cells without inhibitor (Fig. 6c,d). These data suggested a role of TOPII-mediated DSB in enhancing transcriptional elongation by facilitating Pol II pause release. Taken together, our data suggest a novel function for DSB and DDR signalling in transcriptional elongation and the crosstalk between DDR signalling and transcriptional elongation at stimulus-inducible genes in humans.

## Discussion

Our findings provide evidence indicating that DNA breaks and DDR signalling occurs during Pol II pause release and processive elongation at inducible genes. Heat-shock and oestrogen receptor engagement were previously reported to induce γH2AX foci accumulation in mammalian cells^24^,^43^, and DNA repair enzymes have been implicated to play roles in pluripotency and transcription^27^. Providing an insight to these reported observations, our data here propose a new mechanism suggesting that DDR signalling is a positive element for productive transcription. DNA damage-induced signalling in the ‘gene body' during Pol II elongation is an important finding in addition to previously reported DNA strand breaks in the promoter region upon nuclear receptor gene activation^25^,^26^, and suggests that DNA topological modification may be required for both transcriptional initiation and elongation.

We propose that signals originating from transcriptionally induced promoters activate ATM and DNA-PK, and thus play an important role in regulating the checkpoint transition between the early elongation (Pol II pausing) and processive elongation. It is not clear from the present study how these DDR kinases could be recruited or activated during transcription. However, a study has shown that nuclear receptors could recruit a large complex containing DNA-PK, Ku70 and 80, ATM and PARP1 to activated genes^25^,^41^. Huang *et al*.^44^ have also reported direct binding of HSF1 to DNA-PK to stimulate its kinase activity. Another study by Ebmeier and Taatjes^45^ reported that activated mediator complexes, associated with a transcriptional activator, SREBP, bind to ATM, ATR and DNA-PKcs. These results suggested direct or indirect interactions between upstream (promoter) activators and downstream (TSS, pausing site) DNA damage signalling upon transcriptional activation.

We consider the transcriptional regulatory mechanism proposed in our studies to be novel, and deployed in parallel with the established P-TEFb pathway, modulating the DNA topological state or transcriptional microenvironment, while P-TEFb regulates the activities of protein factors such as Pol II, NELF and DSIF. Supporting the role of DDR signalling to modulate DNA architecture, topoisomerases and DNA repair enzymes such as XPG have been proposed to induce permissive chromatin structures and chromosome bending to facilitate transcriptional activation at some genes^20^,^25^,^27^,^46^. One might wonder at the extent of DNA breaks likely to be involved when transcriptional activation, such as heat shock or immediate-early responses, is initiated. However, many cells seem to possess a remarkable ability to repair and seal multiple broken DNA pieces with processivity and dependable precision: for example, there are about 50 million Okazaki fragments per a cell cycle in mammalian cells, the vast majority of which become repaired^47^.

It is known that negative and positive supercoiling are generated in the upstream and downstream of the elongating Pol II complex^17^,^48^. For negative supercoiling, R-loops are often formed, particularly in the TSSs of housekeeping genes^18^,^49^. Topoisomerase I, Aquarius and XPG have been reportedly involved in resolving R-loop^18^,^50^. Interestingly, a recent study has shown that topoisomerase I along with DNA repair enzymes is recruited to activated enhancer RNAs, implying a potential R-loop involvement in enhancer RNA transcriptional activation^51^. Although less extensively studied, the positive supercoiling generated downstream of the elongating Pol II complex could stall Pol II at the pausing site as recently proposed *in vitro*^17^. If this DNA torsion by positive supercoiling is one of the stabilizing elements of Pol II pausing, DNA strand breaks generated in the proximity of paused Pol II could be a straightforward solution to release the torsion. Indeed, both topoisomerase type I and II appear to be recruited to the genes that are highly activated (Supplementary Fig. 7)^51^,^52^. This may imply the functions of topoisomerases to resolve the negative and positive supercoiling during transcriptional activation and elongation. In addition, a role for DNA strand breakage in Pol II elongation could also explain why more genomic mutations have been found in the actively transcribed genes^21^. Supporting these proposals, our studies visualized a DNA break locus on the template DNA during processive elongation and fragility of the non-template DNA at the Pol II pausing site of a model paused gene, *HSPA1B*. Our study provides strong evidence of onset of DNA breaks and DDR in the TSSs and gene bodies of many transcribing paused genes and the requirement of these events for effective transcriptional elongation. These findings also imply the significance of strand breaks and concurrent repair associated with transcriptional elongation.

**Figure 7:**
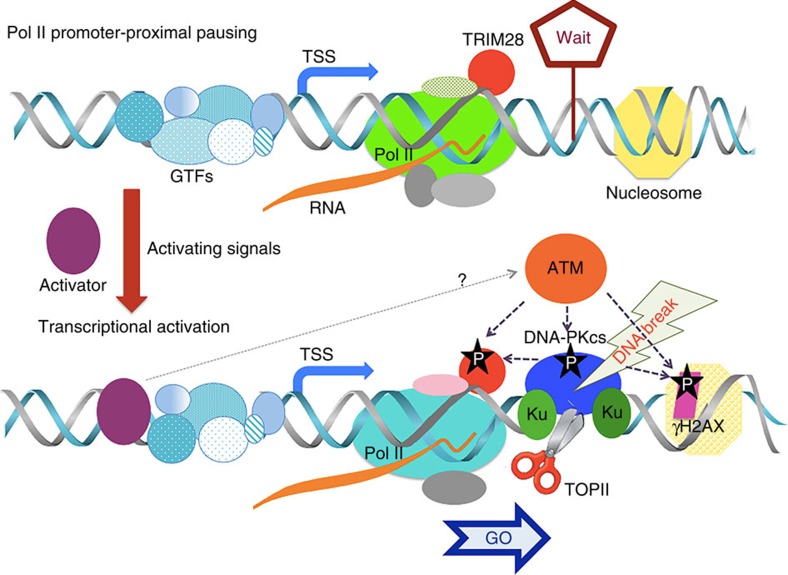
Model of DNA break and DDR signalling for Pol II pause release and elongation. During the uninduced state (top), Pol II is paused at the promoter-proximal site in which TRIM28 stabilizes the pausing. Upon transcriptional activation (bottom), DNA breaks occur in the downstream of TSS and gene body and DDR signalling intermediate proteins become rapidly activated and associated with transcriptional elongation in stimulus-inducible genes.

We therefore propose a novel mechanism for transcriptional regulation involving coupling between DDR signalling and transcriptional elongation. In this model, Pol II pause release and processive elongation are associated with TRIM28, γH2AX and DNA-PK phosphorylation events dependent on a pathway involving the key DDR kinase ATM. These activated intermediates appear to be associated with elongating Pol II. We further propose a key role for topoisomerase II, an enzyme that may mediate DNA strand breaks and exert important topological effects on DNA during Pol II pause release and transcriptional elongation (Fig. 7).

## Methods

### Cell culture and experimental conditions

HEK293 cells (obtained from American Type Culture Collection) in the study were grown in DMEM supplemented with 10% fetal bovine serum (FBS) and 1% penicillin/streptomycin (P/S) solution. For ChIP–PCR ± heat-shock assays, HEK293 cells grown in a dish were heat-shocked in a water bath at 43 °C for assigned time durations and were crosslinked in 0.75% formaldehyde followed by quenching crosslinking with glycine in a final concentration of 125 mM to media. To test kinase inhibitors for γH2AX/TRIM28 phosphorylation, the cell culture was exchanged with a fresh warm medium including a desired inhibitor (or DMSO for control) an hour before heat shock. As a general rule, media (with or without an inhibitor) was preheated to 43°C to be supplied to cells starting heat-shock induction. Cells were rinsed with cold PBS twice before scraping them for next steps or snap freezing at −80. Inhibitors used in this study are as follows: NU7441 from Tocris Bioscience (Cat. No 3712) and KU55933 from Abcam (ab120637). Stock solutions (1,000 × ) were made dissolving the inhibitors as 2 mM and 10 mM in DMSO to target the final concentrations of 2 μM and 10 μM for NU7441 and KU55933, respectively. For serum induction experiments, HEK293 cells were grown to about 80% confluence. The cells were incubated in DMEM including 0.1% FBS and 1% P/S solution for 17.5 h and then induced by serum by incubating in DMEM supplemented with 18% FBS and 1% P/S solution. After serum induction, cells were collected at corresponding time points listed in figures. For inhibition experiment, HEK293 cells were incubated in the 0.1% serum media for 17.5 h. The media was exchanged with the 0.1% serum media with ICRF193 (Sigma I4659-1MG), NU7441 (Tocris Bioscience Cat. No 3712) or Flavopiridol (Sigma F3055) at the final concentration of 10, 2 and 1 μM in 0.1% DMSO, respectively. The cells were incubated with an appropriate inhibitor for 1 h (15 min or 1 h for ICRF193) before serum induction with 18% serum media including the inhibitor in the same concentration described above in 0.1% DMSO for 15 min. Control cells were prepared side by side using DMSO only at the final concentration.

### Chromatin immunoprecipitation and PCR

ChIP–PCR experiment was conducted as described^12^. Antibodies used in immunoprecipitation (IP) were TRIM28 antibody from Bethyl A300-274A (5 μg per IP), phospho-TRIM28 (S824) antibody from Bethyl A300-767 A (5 μg per IP), total Pol II antibody from Santa Cruz Biotechnology sc-899 (5 μg per IP), γH2A.X (phospho S139) antibody from Abcam ab2893 (5 μg per IP), pDNA-PK_CS_ (Thr 2609) antibody from Santa Cruz Biotechnology sc-101664 (3.5 μg per IP), Ku70 (A-9) antibody from Santa Cruz Biotechnology sc-5309 (3 μg per IP), S2 phosphorylated Pol II antibody from Abcam ab5095 (5 μg per IP) and Topoisomerase II beta antibody from Abcam ab58442 (3 μg/IP).

### Comet assay

Control and serum-induced HEK293 cells were washed with cold PBS and collected by scraping. The Comet assays were performed using COMET SCGE kit (Enzo Life Sciences) and following the protocol included in the kit. We opted the electrophoresis in TBE buffer rather than denaturing buffer. Dried samples were stained using silver (TREVIGEN 4254-200-K) or SYBR GREEN (Life Technologies) following the manufacturer's instructions.

### Immunofluorescence

Approximately, 2 × 10^5^ HEK293 cells were seeded in each well of a four-well chamber slide and cultured for 24 h in serum-containing medium. Subsequently, cells were switched to 0.1% serum-containing medium for 17.5 h and then to 18% serum-containing medium for 5–15 min. Immunostaining was carried out as described previously^53^. Briefly, the cells were fixed with 4% paraformaldehyde for 20 min at room temperature and permeabilized with 0.5% Triton X-100 in PBS while on ice for 10 min. Nonspecific binding sites were blocked with 5% goat serum in 1 × PBS for 60 min at room temperature before probing with pDNA-PKcs^54^ or pTRIM28 (A300-767A, Bethyl) primary antibodies (1:500 dilution) for 3 h at room temperature. Cells were then washed three times with 1% bovine serum albumin (BSA) and incubated with the appropriate secondary antibodies diluted in 1% BSA and 2.5% goat serum for 1 h. Secondary antibodies (anti-mouse or anti-rabbit) conjugated with Alexa Fluor 488/555 were purchased from Invitrogen. Subsequently, the slides were washed five times with 1% BSA for 5 min each and the nuclei were counter stained with 4,6-diamidino-2-phenylindole (DAPI; Vector shield). For γH2AX, HEK293 cells grown on coverslips after treatment were fixed with 4% paraformaldehyde for 10 min and permeabilized with 0.1% Triton X-100 for 5 min at room temperature. Cells were stained with anti-mouse-γH2A.X primary antibody (ab26350, Abcam) for 1 h at room temperature. Cells were then washed three times with PBS (1 × ) and then further incubated with goat anti-mouse Alexa 488 (green) antibody along with DAPI (blue, nuclear staining).

### Image acquisition

Images were captured using an LSM 510 Meta laser scanning confocal microscope with a × 63 1.4 numerical aperture Plan-Apochromat oil immersion objective as described previously^55^. Images were taken at *z*-sections (24 sections) of 0.35-μm intervals using the 488- (Alexa 488), 543- (Alexa 555) and 405-nm (for DAPI) lasers. The tube current of the 488-nm argon laser was set at 6.1 A. The laser power was typically set to 3–5% transmission with the pinhole opened to 1–2 Airy units. Subsequently, the *z*-sections were assembled using the Imaris software (Bitplane) and then used for further analysis. For γH2AX, mounted coverslips with stained cells were later analysed using a Zeiss LSM 510 (metaconfocal) microscope using numerical aperture 1.4, × 63 oil immersion objective set with the pinhole set at 0.9–1.0 Airy units (Carl Zeiss, Jena, Germany). Fluorophores were visualized using the following filter sets: 488-nm excitation and band-pass 505–530 emission filter for Alexa 488; 543-nm excitation and long pass 560–615 filter for Cy3. DAPI was visualized using 405-nm excitation and 470–500-nm emission band-pass filters.

### Primer extension

HEK293 cells were heat-shocked at 42 °C for 0 or 5 min and washed once with cold 1 × PBS before being collected by scraping. Genomic DNA was prepared using PureLink Genomic DNA Mini Kit (Life Technologies). Each primer was labelled with [α-^32^P] dATP using T4 DNA polynucleotide kinase (New England Biolabs). For primer extension, 1 μg of genomic DNA was mixed with about 4.5 × 10^6^ c.p.m. per sample of labelled primer, 10 μM of each dNTP and thermostable, non-proofreading Tag DNA polymerase (1 unit, Invitrogen) in 25 μl of 10 mM Tris-HCl (pH 8.3), 50 mM KCl and 1.5 mM MgCl_2_. PCR cycles are 30 cycles of 1 min 20 s at 95/60/72 °C, followed by a final extension for 15 min at 72 °C. PCR products were separated on denaturing polyacrylamide gels and exposed to X-ray film. The primer sequences are available in Supplementary Table 1.

### RT–qPCR

HeLa cells were starved in 0.1% serum for 17.5 h and then stimulated with 18% serum for indicated time durations. Total RNA samples were collected and reverse transcribed into complementary DNA using OneTaq RT-PCR Kit (New England Biolabs). Real-time PCR was performed in CFX96 Real-time PCR detection system (Bio-Rad) using β-actin as an internal control. The result presents mean±s.d. after normalization by sham (no serum) group. The primer sequences are available in Supplementary Table 1.

### ChIP-seq

ChIP and DNA preparation were performed as described above and in ref. 12. Illumina libraries were constructed using Kapa Biosciences Illumina library prep kits according to the manufacturer's protocol or as described in ref. 12. Samples were quantified by qPCR and pooled at equal ratio before sequencing on the Illumina HiSeq2000 or HiSeq2500 sequencing platform. The resulting short reads were aligned against the hg19 reference Human genome. ChIP-seq data processing was performed as following. Sequencing FASTQ files for all samples were aligned to the hg19 reference human genome (Ensembl GRCh37.75) using BWA aligner^56^. Following alignment, duplicate reads were filtered using Picard Tools and the remaining reads were filtered to only include primary alignments. Alignment files for biological replicates were subsequently sorted and merged using SAMtools^57^. To generate a list of tag/read-enriched regions along the genome, we use the peak-finding algorithms as part of the HOMER suite of analysis tools^58^. Background tag distributions are determined empirically from the matched input samples in each condition. We use the default parameters for the peak-finding algorithm, setting the threshold at fourfold enrichment over background with a corresponding Poisson-based false discovery rate value of 0.001. Following peak determination, we cross-referenced protein-coding genes (as annotated by the Ensembl GTF file provided with the GRCh37.75 reference) with those genomic regions determined to be enriched for sequencing tags. The pausing index (described below) was subsequently calculated for all annotated primary transcripts of these genes. Read quantifications were performed using HTSeq^59^ and only included those reads mapping to a single gene. To account for sequencing library size differences, we linearly scale all counts by a factor (≤1) related to the ratio of total mapped reads in the respective alignment files. Thus, the effective number of mapped reads in each library is scaled to match that of the alignment file with the fewest reads and the relative abundance of Pol II in each condition is available. Finally, a single primary transcript was selected for each gene based on the criteria of maximum body-read density (reads per base pair contained between +250 to the end of transcript). Further, we filter out those genes/transcripts that do not exceed a threshold body-read density of 0.05. This threshold is arbitrary but yields an adequate number of genes for downstream analyses. The pausing index attempts to quantify net movement of Pol II from the TSS region into the gene body and is defined as the ratio of read density in the TSS-proximal region to that of the gene body. In this study, the TSS-proximal region was defined to be (−150, +150) about the annotated TSS and the gene body is defined as +250 to the end of the specific primary transcript. We note that this quantity is sensitive and highly variable in those genes with low Pol II occupancy; removing such genes via filtering at a threshold read density partially mitigates this issue. To calculate the empirical confidence intervals, a bootstrap resampling procedure was employed. A total of 10^5^ random samples were drawn from the respective empirical distributions and the effect size is calculated as the difference between the medians of the resampled distributions. The centred 95% empirical confidence interval was calculated from the resulting distribution of effect sizes. Under the null hypothesis that the effect size is positive, the empirical *P* value is taken as the percentage of random samples where the effect size was negative (except where noted).

## Additional information

**Accession codes:** The ChIP-seq data has been deposited into NCBI Gene Expression Omnibus under accession number GSE75170.

**How to cite this article:** Bunch, H. *et al*. Transcriptional elongation requires DNA break-induced signalling. *Nat. Commun.* 6:10191 doi: 10.1038/ncomms10191 (2015).

## Supplementary Material

Supplementary Information Supplementary Figures 1-10 and Supplementary Table 1
