# Supplementary material for: Transcriptional elongation requires DNA break-induced signalling
Source: Nat Commun. 2015 Dec 16;6:10191. doi: 10.1038/ncomms10191 (PMC4703865; doi:10.1038/ncomms10191)
Supplement: Supplementary Information — Supplementary Figures 1-10 and Supplementary Table 1 [file ncomms10191-s1.pdf]

## Supplementary Figures

a.

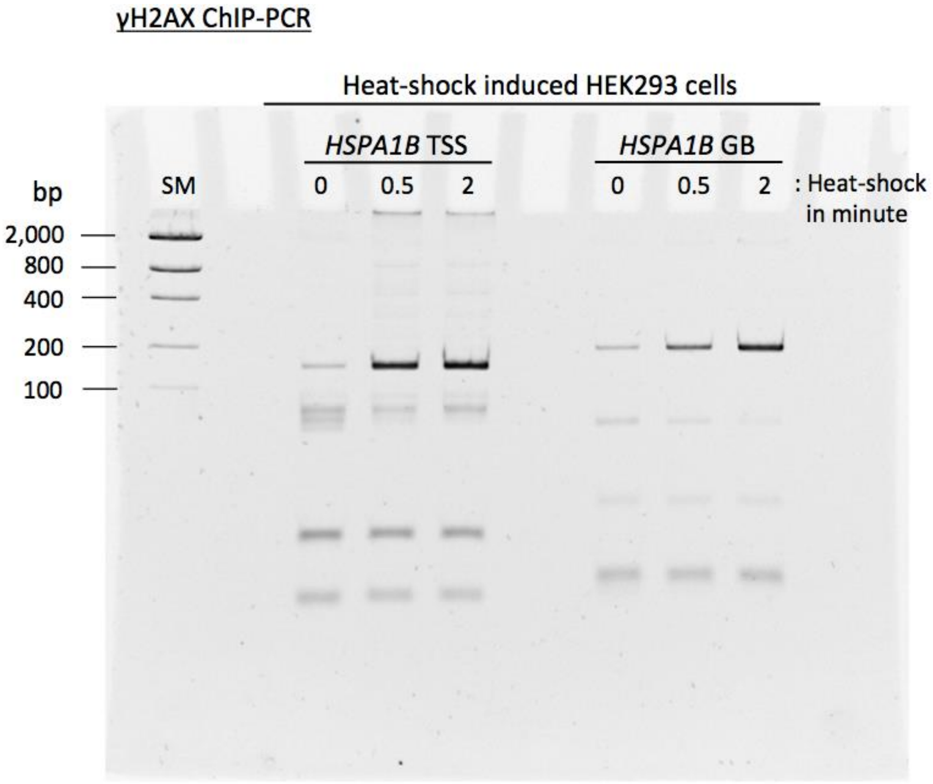


b.

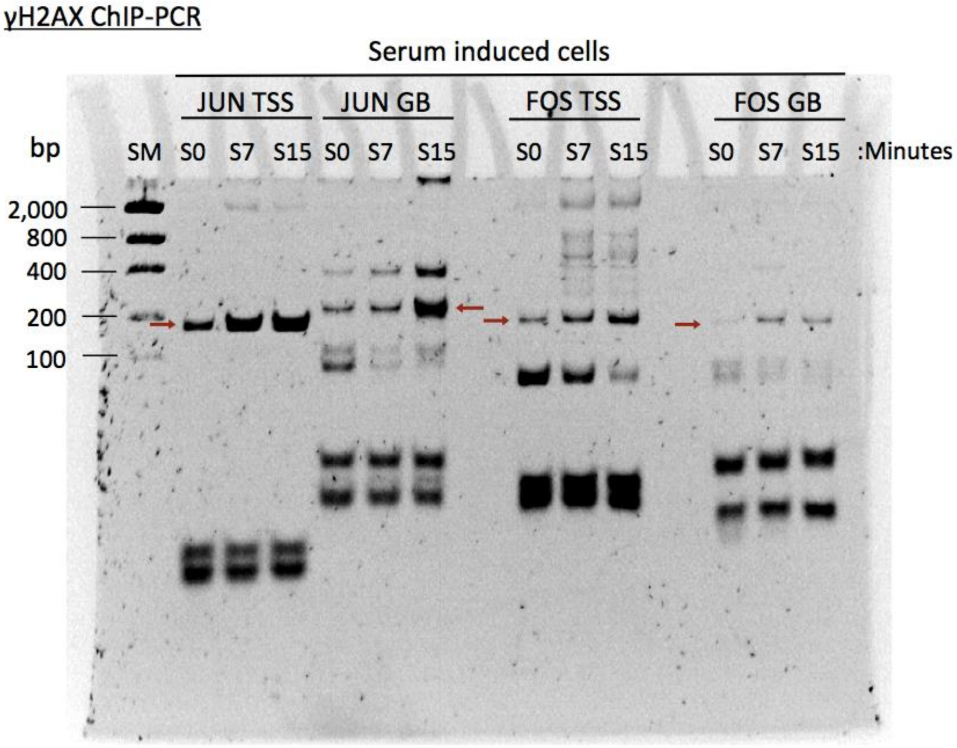

c.

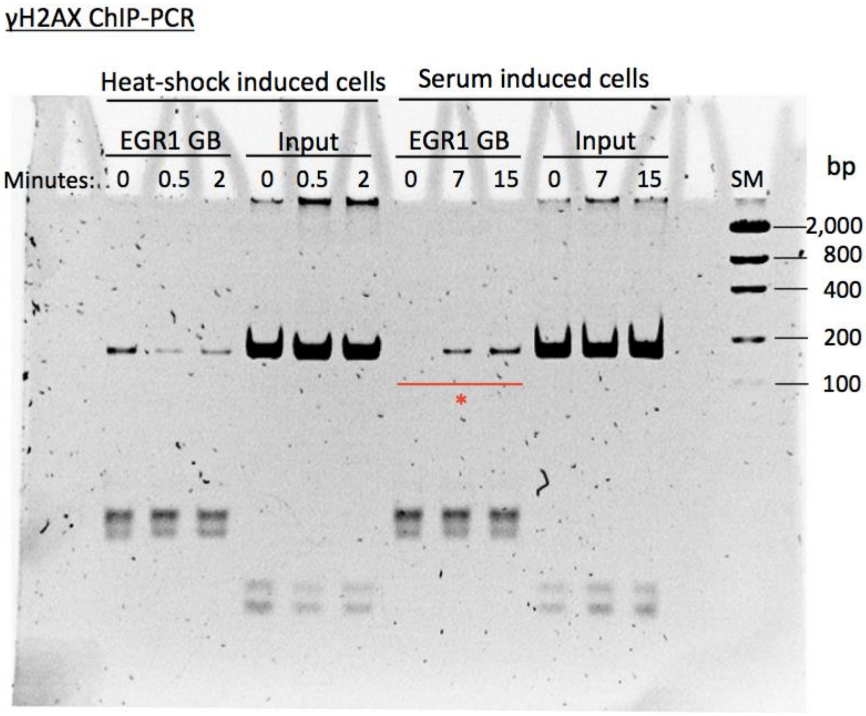


**Supplementary Figure 1. ChIP-qPCR analysis of γH2AX at paused genes such as *HSPA1B* and immediate early genes upon transcriptional activation.** (**a**) Heat shock induced transcriptional activation of HSPA1B recruits γH2AX on the gene in the TSS and gene body (GB). SM, size marker; 0, non heat shock control; 0.5, 30 sec heat shock; 2, 2 min heat shock. (**b**) Serum induction accumulates γH2AX at the TSS and GB of *JUN* and *FOS* genes. SM, size marker, S0, control; S7 and S15, serum induced for 7 and 15 minutes, respectively. (**c**) γH2AX in the gene body of *EGR1* gene after heat shock (negative control) and serum induction. The level of γH2AX protein became increased upon serum-induced transcriptional activation. Contrast to serum induction, the level of γH2AX displayed no increase with heat-shock.

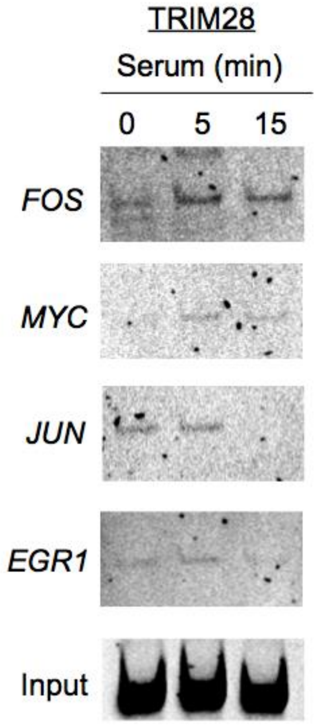


**Supplementary Figure 2. ChIP-qPCR analysis of TRIM28.** Contrast to phospho-TRIM28, the level of total TRIM28 displayed no increase at immediate-early genes as *FOS*, *MYC*, *JUN*, and *EGR1* upon serum induction.

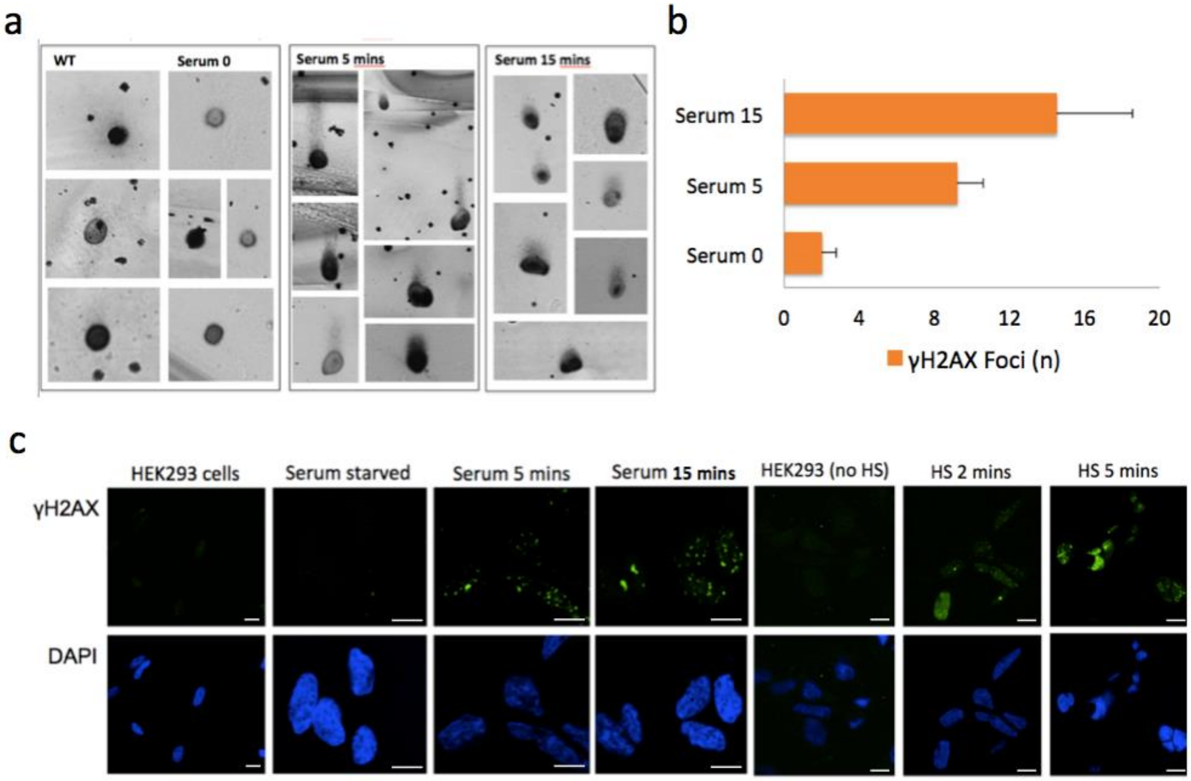


**Supplementary Figure 3. DNA break occurrence upon serum induced transcriptional activation.** (**a**) Comet assay followed by Silver staining of cells visualized the tail formation behind cell migration suggesting DNA break event upon serum induction. WT HEK293, Wild Type HEK293 cells; Serum 0, serum starved for 17.5 hours; Serum 5 min or 15 min, serum induced for 5 or 15 minutes after serum starvation for 17.5 hours. (**b**) γH2AX foci quantification. A bar graph showing average numbers of γH2AX foci with standard deviation in serum starved (serum 0) and serum induced HEK293 cells (serum 5 and serum 15; n= 10 cells). (**c**) Cells were targeted to activate multiple genes using serum induction for 5 (Serum 5 min) and 15 minutes (Serum 15 minutes) after serum starved for 17.5 hours (Serum starved) or through heatshock for 0 (no HS), 2 (HS 2 mins), or 5 minutes (HS 5 mins). γH2AX was increased in serum-induced or the heat-shocked cells compared to non-stimulated controls (a scale bar = 10 μM).

a.

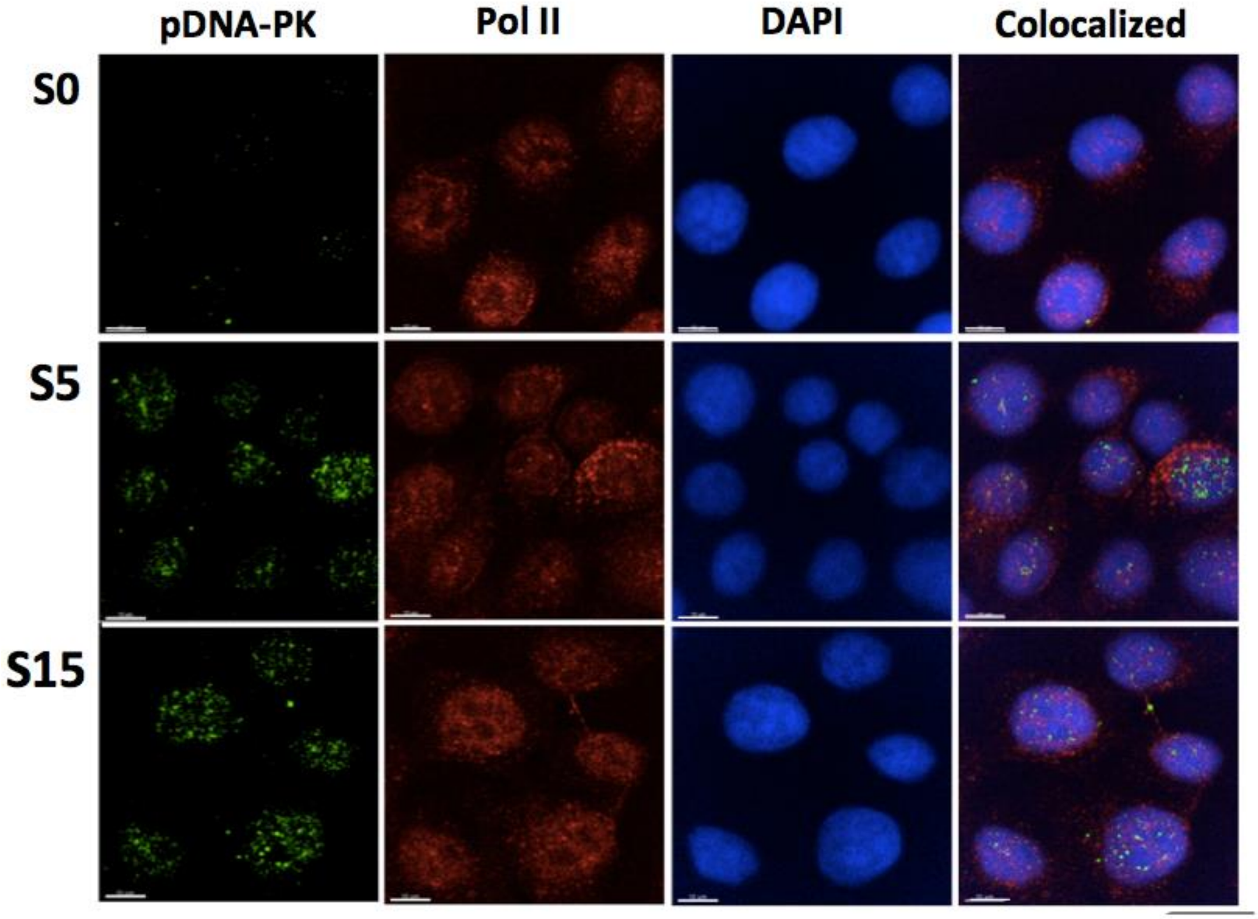

b.

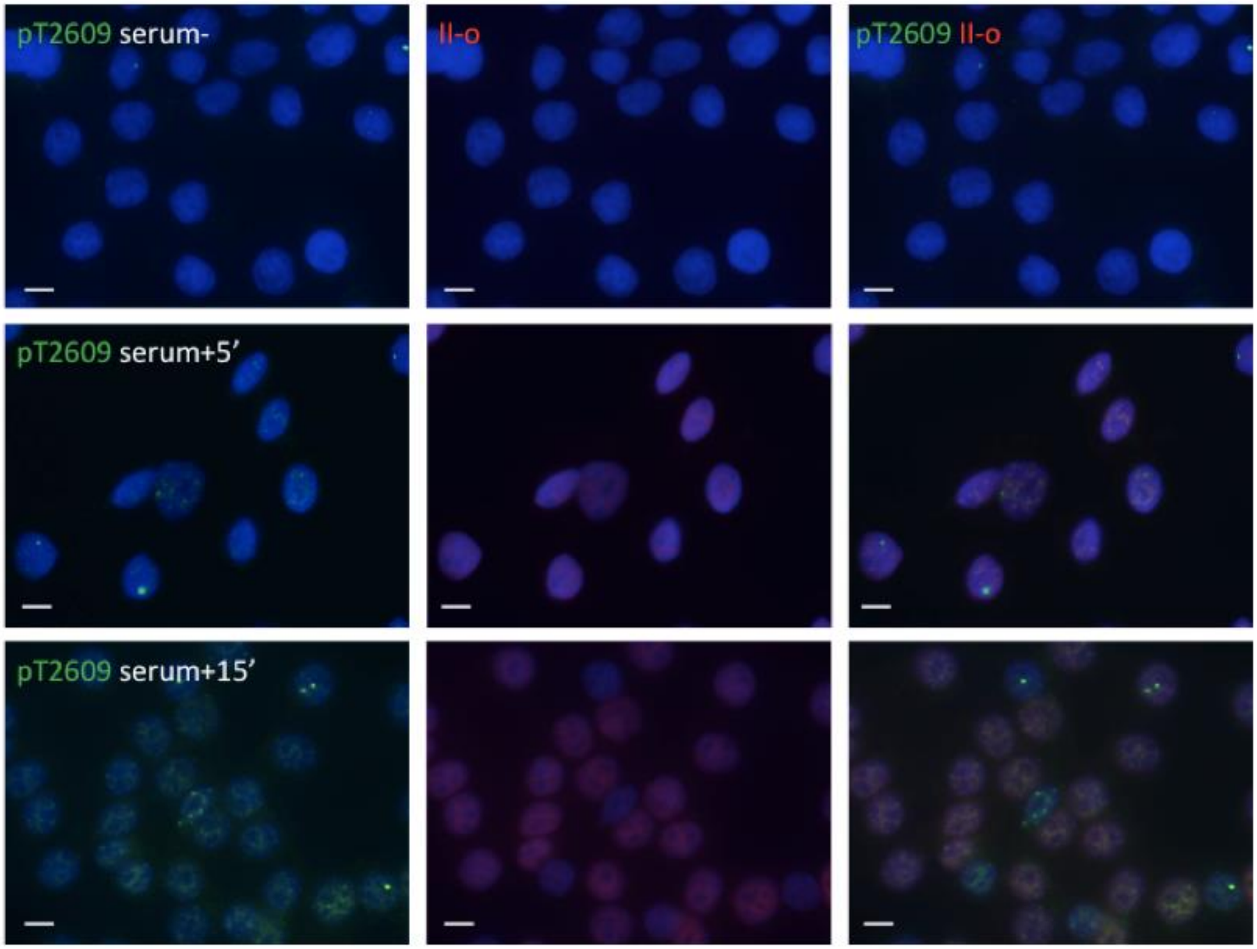

c.

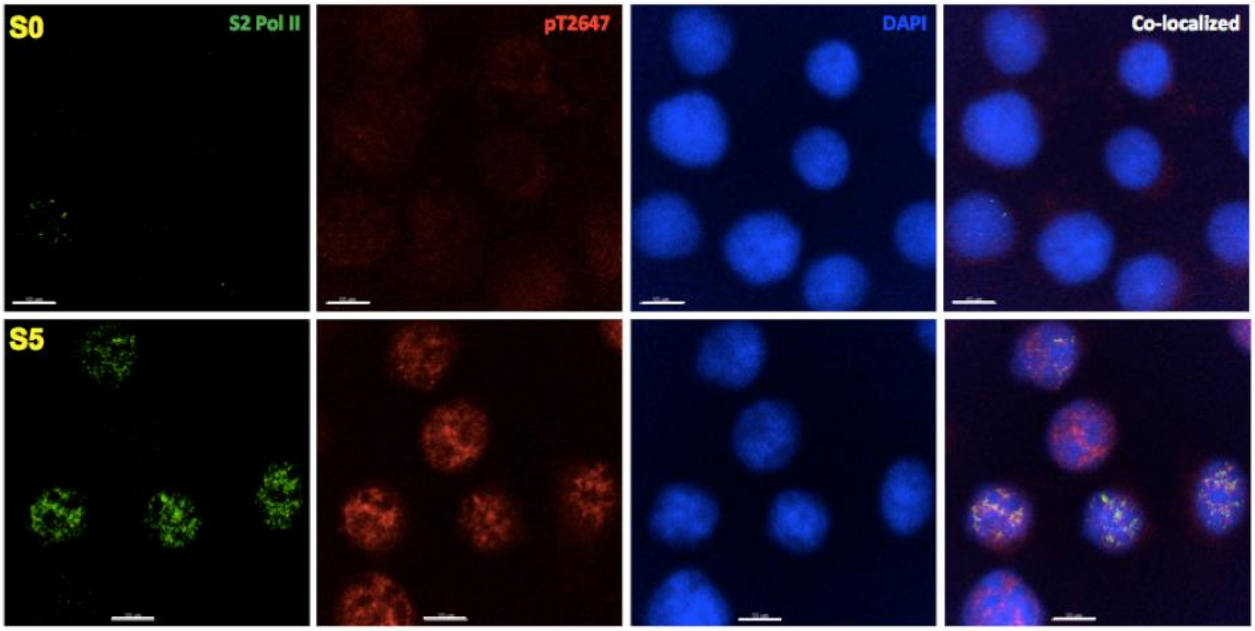


**Supplementary Figure 4. Immunofluorescence of activated DNA-PKs in HEK293 cells.** (**a**) Phospho-DNA-PK (T2609) and unphosphorylated Pol II. Contrast to S2 Pol II that becomes enriched in serum induced cells for transcriptional activation, total Pol II is found in both serum un-induced and induced cells and mildly co-localized. (**b**) Phospho-DNA-PK (T2609) and S2 Pol II (II-o) showing increased levels and co-localization in serum-induced HEK293 cells. (**c**) Another signature ATM-dependent phosphorylation site of DNA-PK, residue T2647 becomes phosphorylated and co-localized with S2 Pol II upon serum induction. Serum induction for 5 minutes (S5) after serum starved for 17.5 hours (S0). A scale bar, 10 μM.

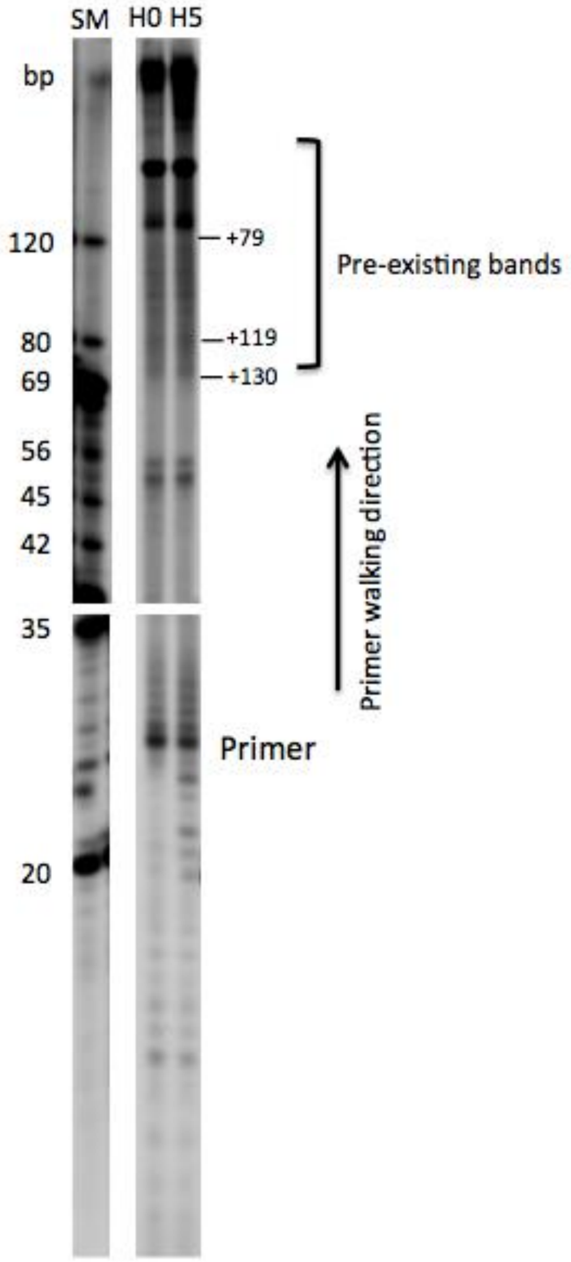


**Supplementary Figure 5. Primer extension analysis to map DNA break loci in the TSS of the non-template *HSPA1B* DNA.** A reverse primer that extends from +198 was used to identify the DNA break locus on the *HSPA1B* non-template DNA. Numerous DNA bands, marked with a bracket, appeared in the non-template DNA in both not activated (H0) and heat-shock activated (H5) *HSPA1B*. This may imply susceptibility of the *HSPA1B* non-template DNA for DNA break. SM, Size marker in base pairs. Locations from TSS on the non-template DNA were marked on the right side of the image.

a.

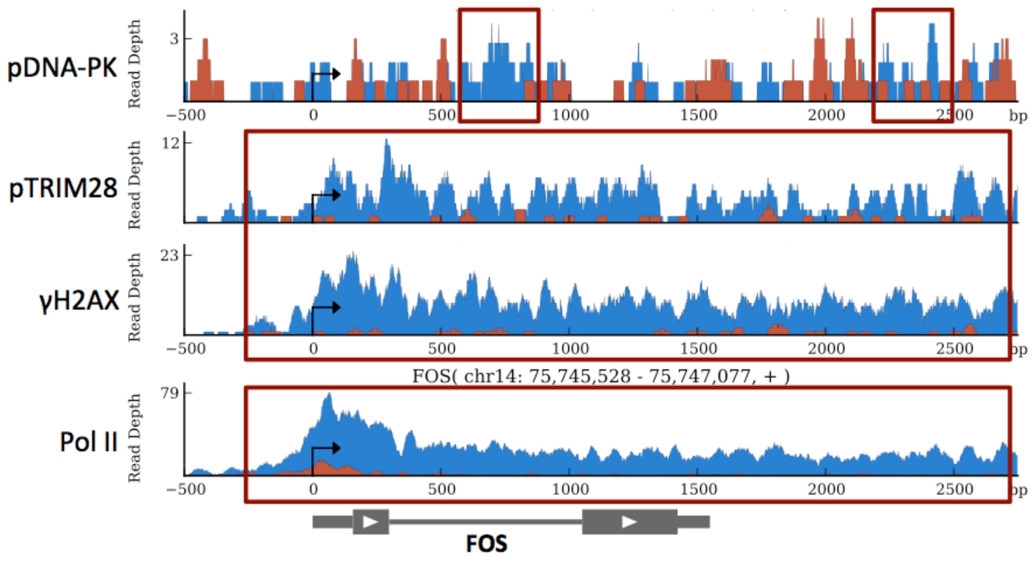


b.

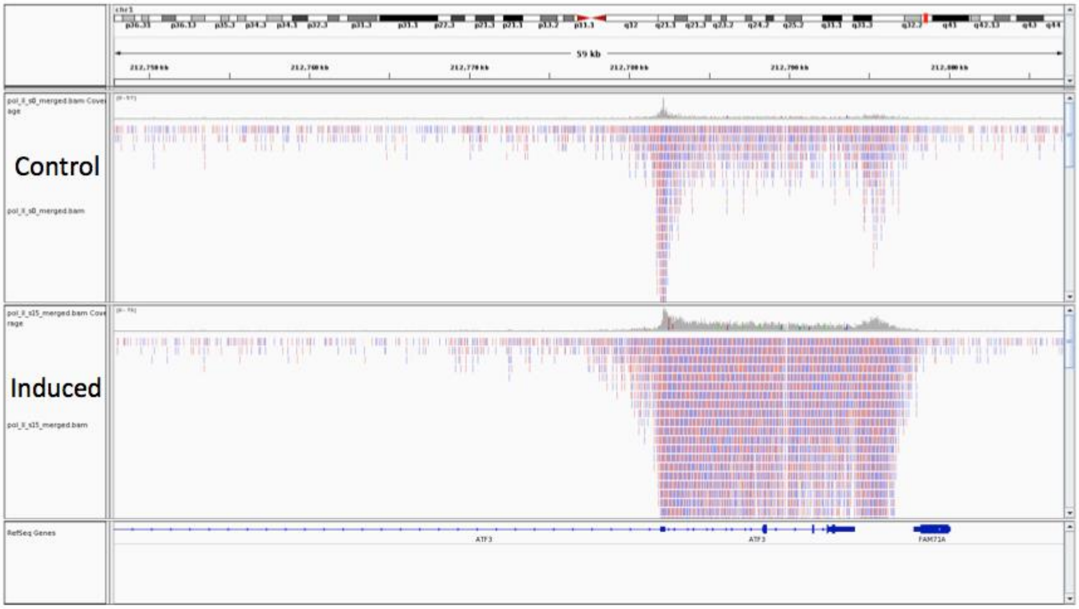


**Supplementary Figure 6. ChIP-seq analysis of Pol II and DNA repair proteins at *FOS*, an immediate early gene and of Pol II at *ATF3*.** (**a**) Chromosome viewers of Pol II, γH2AX, pTRIM28 (S824), and pDNA-PK (T2609) at a representative immediate early gene, *FOS*. Blue peaks for serum induced cells and red peaks for control, non-serum induced cells. Peaks appearing upon serum induction were marked with red boxes. (**b**) Chromosome viewers of Pol II at *ATF3*, a mitogen/serum early response gene.

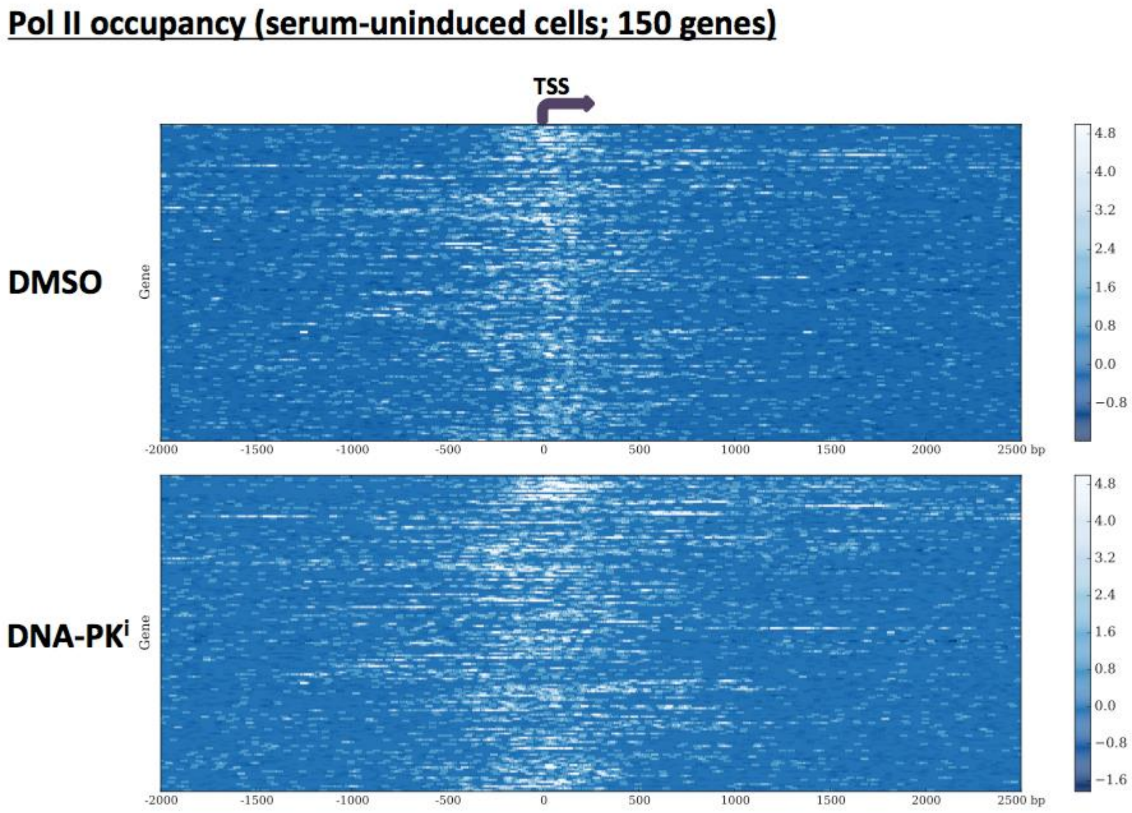


**Supplementary Figure 7. ChIP-seq analysis of Pol II occupancy in serum-inducible genes with or without DNA-PKcs inhibition**. Pol II occupancy became increased in the TSSs of a subset of serum-inducible genes (n= 150) with a DNA-PKcs inhibitor, NU7441 (DNA-PK$^{i}$) in non-serum induced HEK293 cells.

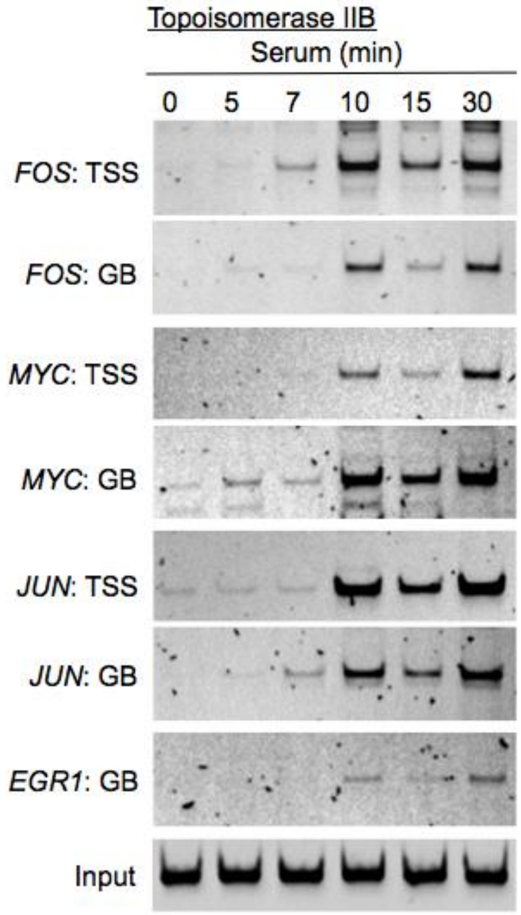


**Supplementary Figure 8. ChIP-qPCR analysis of topoisomerase IIβ at representative immediate early genes.** Topoisomerase IIB occupancy was increased at *FOS*, *MYC*, and *JUN* in the TSS and gene body (GB) and *EGR1* in GB upon serum induction.

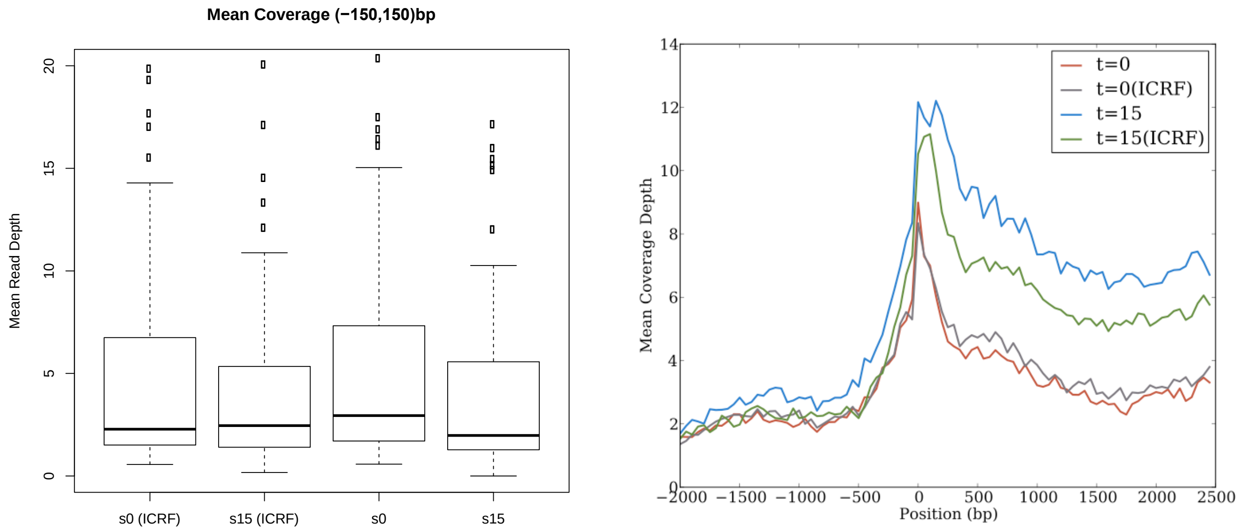


**Supplementary Figure 9. Pol II occupancy changes with or without a DSB inhibitor. Left:** A box plot showing Pol II occupancy changes at TSSs of a subset of serum activated genes (n= 109). Unlikely to the large decrease in Pol II gene body occupancy upon serum induction in the presence of ICRF (topoisomerase II inhibitor, Fig. 4C), Pol II occupancy was slightly increased at TSSs upon serum induction with the inhibitor. **Right:** Metagene analysis of Pol II to show a reduced Pol II occupancy in the gene body of a subset of serum activated genes (n=109) upon induction in the presence of ICRF193, a TOPIIB inhibitor to prevent from DSB. Control, non-induced cells, t=0; non-induced cells with ICRF193, t=0(ICRF); induced cells, t=15; and induced cells with ICRF193, t=15(ICRF).

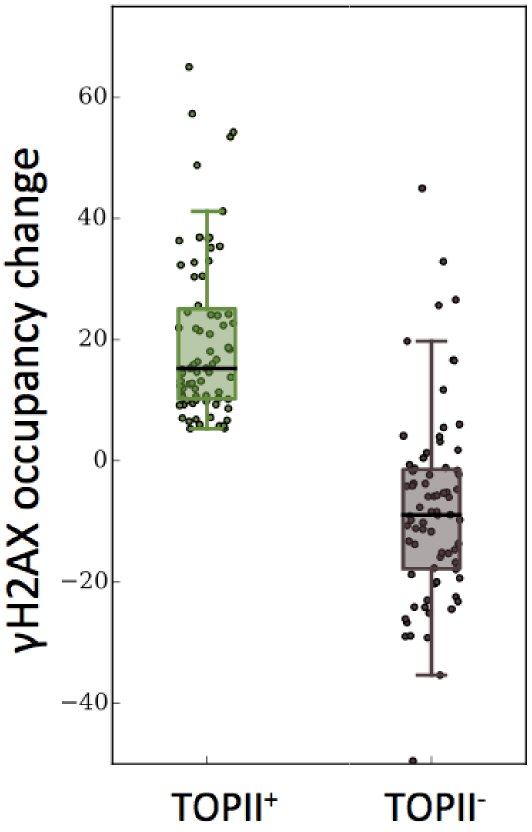


**Supplementary Figure 10. Topoisomerase II induces DNA breaks during transcriptional elongation.** ChIP-seq analysis of γH2AX with ($TOPII^-$) or without topoisomerase II inhibitor ($TOPII^+$) showed the function of topoisomerase II in transcriptional elongation and DDR signaling. Box plots were generated with serum-activated genes with increased γH2AX in DMSO control (n= 75).

# Supplementary Table 1

## Supplementary Table 1. The sequences of oligos and primers used in this study

| | |
|---|---|
| **For ChIP-PCR Assay** | |
| **HSPA1B forward from -167** | GCGGCACCCTGCCCTCTGATTGGTCCAAGGAAGGC |
| **HSPA1B reverse from +10** | GCCGTTTTCCGGACCGCGCGCCCCTCGGC |
| **HSPA1B forward from +61** | CCGCGGTCCCAAGGCTTTCCAGAGCGAACCTGTGCG |
| **HSPA1B reverse from +198** | GGGCTCCGCTCTGAGACTGGGGGCTGG |
| **HSPA1B forward from +163** | CCGCCGTTTCCAGCCCCCAGTCTCAGAGCGG |
| **HSPA1B reverse from +313** | GGTCGTTGGCGATGATCTCCACCTTGCCGTGTTGGAA CACCCC |
| **HSPA1B forward from +1861** | GGTGTCAGCCAAGAACGCCCTGGAGTCC |
| **HSPA1B reverse from +2010** | CTCCTTCCTCTTGTGCTCAAACTCGTCCTTCTC |
| **FOS TSS Forward** | GAC CGT GCT CCT ACC CAG CTC TGC TCC ACA GCG CCC |
| **FOS TSS Reverse** | GAG TGG TAG TAA GAG AGG CTA TCC CCG GCC |
| **FOS Gene body Forward** | CAC AGA CCC AGG CCT GGC TCA ACA TGC TAC |
| **FOS Gene body Reverse** | CAC CAG GCT GTG GGC CTC AAG GAC TTG AAA GC |
| **JUN TSS Forward** | CCA GCC AGG TCG GCA GTA TAG TCC G |
| **JUN TSS Reverse** | GCC TGG GCA GCA GGG CTC TCC TCC |
| **JUN Gene body Forward** | GCG AGG CTG AGC CTA CAG ATG AAC TCT TTC TGG |
| **JUN Gene body Reverse** | CCA AAT CTC TTA TTT ACA AAC AAC ACT GGG CAG GAT ACC C |
| **EGR1 Gene body Forward** | GCT TTC CCG GCC CAG GTC AGC AGC TTC CCT TCC |
| **EGR1 Gene body Reverse** | CTT TTT CTC CCT TTT CCC TTT CTT TCC CCT TTC CC |
| **MYC TSS Forward** | CGG GGC TTT ATC TAA CTC GCT GTA GTA ATT CCA GCG AGA GG |
| **MYC TSS Reverse** | GCG GGA GGG CTG GGC CAG AGG CG |
| **MYC Gene body Forward** | CGC AGC GCC TCC CTC CAC TCG GAA GGA CTA TCC |
| **MYC Gene body Reverse** | GGC GCT CCA AGA CGT TGT GTG TTC GCC TC |
| **For RT-qPCR analysis** | |
| **JUN-Forward** | CCT CAA CGC CTC GTT CCT C |
| **JUN-Reverse** | TTA CTG TAG CCA TAA GGT CCG CT |
| **FOS-Forward** | CAG GTG GCA CAG CTT AAA CA |
| **FOS-Reverse** | GTT TGC AAC TGC TGC GTT AG |
| **EGR1-Forward** | CTT CAA CCC TCA GGC GGA CA |
| **EGR1-Reverse** | GGA AAA GCG GCC AGT ATA GGT |
| **MYC-Forward** | AAT GAA AAG GCC CCC AAG GTA GTT ATC C |
| **MYC-Reverse** | GTC GTT TCC GCA ACA AGT CCT CTT C |
| **ACTIN-Forward** | GCC GAC AGG ATG CAG AAG GAG ATC A |
| **ACTIN-Reverse** | AAG CAT TTG CGG TGG ACG ATG GA |
| **For Primer extension** | |
| **HSPA1B Forward from +1** | GGAAAACGGCCAGCCTGAGGAGCTG |
| **HSPA1B Reverse from +198** | GGGCTCCGCTCTGAGACTGGGGGCTGG |
| **GAPDH Forward** | AGAAGGCTGGGGCTCATTTG |
